# Cerebrovascular imaging of carotid embolization: Amaurosis fugax and transient ischemic attack in motion

**DOI:** 10.1016/j.jvscit.2025.101915

**Published:** 2025-07-10

**Authors:** Bibhas Amatya, Dipankar Mukherjee

**Affiliations:** a Department of Neurosurgery, University of Maryland School of Medicine, Baltimore, MD; b Division of Vascular Surgery, Inova Fairfax Hospital, Falls Church, VA

**Keywords:** Cerebrovascular, Transient ischemic attack, Carotid endarterectomy, Carotid stenosis

## Abstract

We describe a 65-year-old male ophthalmologist who experienced a brief episode of transient monocular vision loss in his left eye, characterized as a “gray-out” lasting a few seconds. Utilizing his medical expertise, the patient recorded the blood flow in his ophthalmic artery, capturing a rare real-time visualization of embolus migration from the central to peripheral regions. The embolism resolved spontaneously, restoring normal vision. Duplex ultrasound revealed high-grade stenosis in the proximal left internal carotid artery, with a peak systolic velocity of 421 cm/s and an end-diastolic velocity of 188 cm/s, consistent with over 90% stenosis. A computed tomography angiogram confirmed the presence of an ulcerated plaque, attributing the vision loss to emboli originating from the carotid artery. Because of the critical stenosis and risk of further cerebrovascular events, a semiurgent left carotid endarterectomy was performed under regional anesthesia. The procedure was completed without complications, and the patient’s vision remained normal postoperatively. This case highlights the unique real-time documentation of an embolic event in an ophthalmologist who recognized the pathophysiology, emphasizing the importance of timely diagnosis and intervention, particularly carotid endarterectomy, in preventing stroke and irreversible vision loss in patients with high-grade internal carotid artery stenosis.

Stenosis of the internal carotid artery (ICA) due to atherosclerotic plaque formation can result in embolization, commonly affecting upstream vessels such as the ophthalmic artery.^1^ When the ophthalmic artery is embolized, it can cause transient monocular vision loss or a condition known as amaurosis fugax.^2^ Prompt identification and treatment of the underlying vascular condition are critical for preventing irreversible vision loss or more severe cerebrovascular complications.

In this report, we present a rare case of a 65-year-old male ophthalmologist who experienced transient monocular vision loss in his left eye due to emboli from a high-grade stenosis of the proximal internal carotid artery. What makes this case unique is the patient’s ability to document the embolic migration in real-time, providing a rare visual correlation between the embolic event and transient vision loss. The patient’s insight into the condition was responsible for timely action and a successful outcome. This case highlights the importance of early vascular evaluation, prompt surgical intervention, and the potential for patient involvement in advancing clinical observations in ophthalmic vascular events.

## Case presentation

A 65-year-old male ophthalmologist presented with a brief episode of transient monocular vision loss in his left eye, describing it as his “vision going gray” for a few seconds before clearing. The patient, given his medical expertise, recorded the blood flow in the ophthalmic artery, demonstrating significant migration of the embolus from the center to the peripheral regions. The embolus was transient as it was dislodged, allowing restoration of normal blood flow and vision.

**Fig 1:**
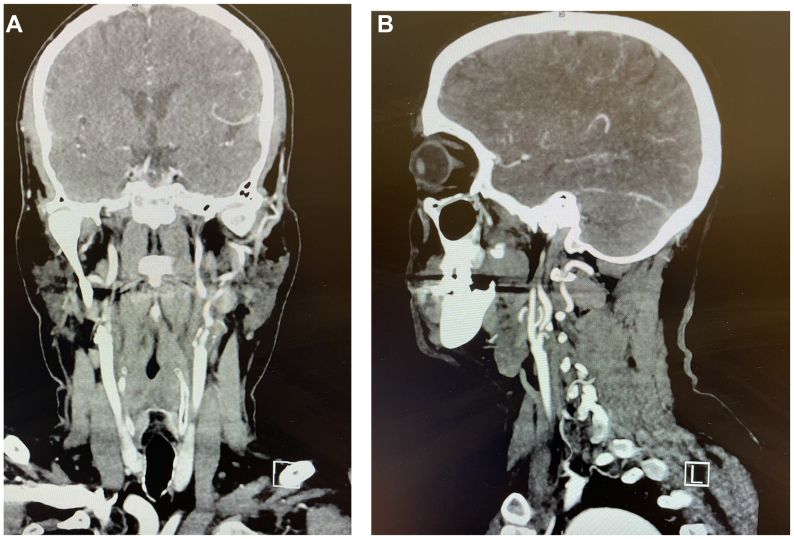
**(A)** This computed tomography angiogram of the neck highlights the high-grade stenosis in the proximal left internal carotid artery (ICA). **(B)** Sagittal view of the significant narrowing of the artery suggesting a high risk of embolic events, which could lead to vision loss in the ophthalmic artery branches.

Further investigation with duplex ultrasound revealed high-grade stenosis in the proximal left ICA, with hemodynamic measurements showing a peak systolic velocity of 421 cm/s and an end-diastolic velocity of 188 cm/s, suggesting over 90% stenosis. A computed tomography angiogram confirmed the presence of an ulcerated plaque in the same region (Fig 1, *A* and *B*). This imaging highlights the high-grade stenosis in the proximal left ICA, where significant narrowing suggests a high risk of embolic events that could lead to vision loss in the ophthalmic artery branches.

**Fig 2:**
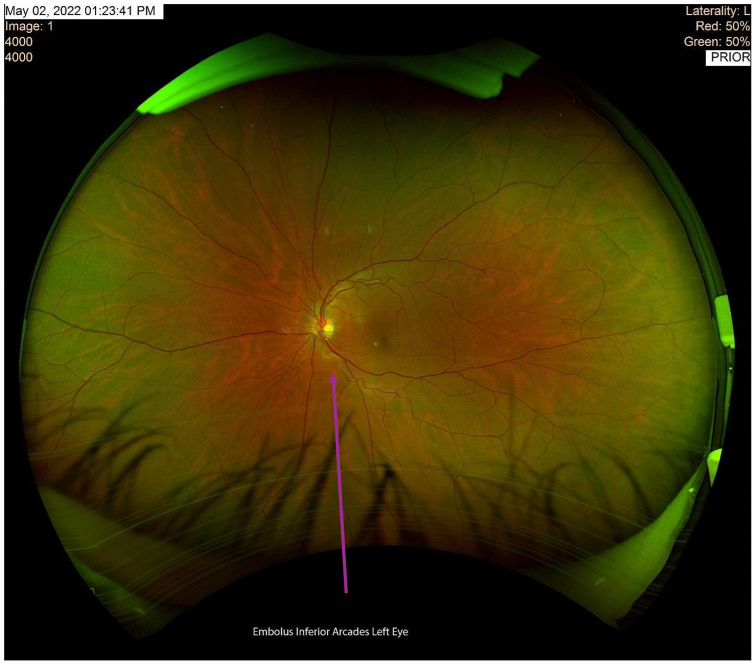
This imaging illustrates the optic nerve head of the patient’s left eye during the episode of transient vision loss.

**Fig 3:**
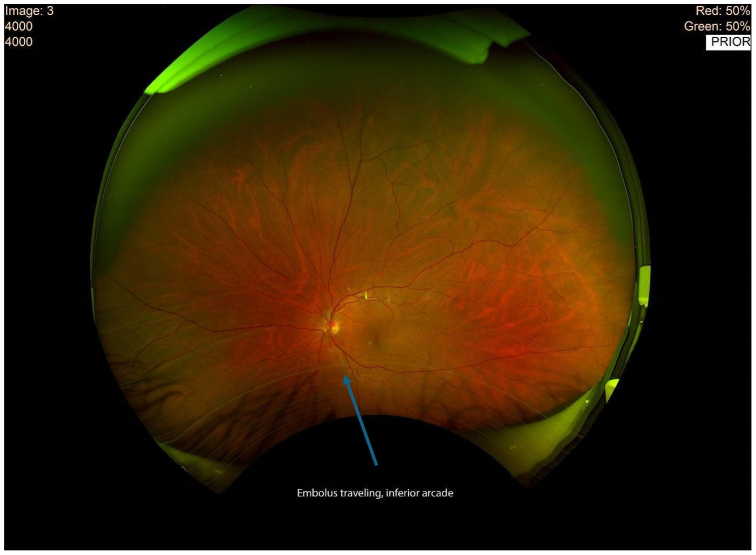
This close-up of the retina shows the embolus as it travels in the inferior branch of the retinal artery, which is responsible for the transient ischemia.

**Fig 4:**
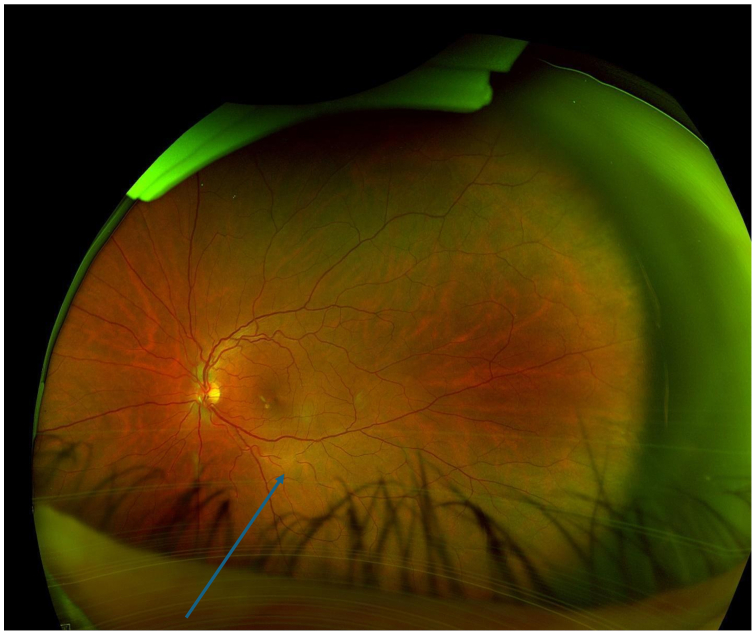
Another close-up of the retina shows the embolus as it travels farther in the inferior branch of the retinal artery.

The patient utilized his medical expertise to record the embolic migration in real time, capturing the embolus within the ophthalmic artery. Imaging of the optic nerve head during the episode of transient vision loss revealed significant ischemic changes (Fig 2). Subsequent close-ups of the retina provided visual confirmation of the embolus as it traveled through the inferior branch of the retinal artery (Figs 3 and 4).

Because of the critical nature of the stenosis and the risk of future cerebrovascular events, a semiurgent left carotid endarterectomy was recommended. The patient consented to the procedure, which was performed under regional anesthesia. After standard preparation, an incision was made along the left sternocleidomastoid muscle, and the carotid sheath was opened, exposing the common, internal, and external carotid arteries. These vessels were controlled with loops, and the patient was anticoagulated with 7000 units of intravenous heparin. The external and common carotid arteries were temporarily occluded. Carotid occlusion was well-tolerated by the patient, who remained alert and responsive throughout the procedure. No shunting was required. Carotid endarterectomy with bovine pericardial patch angioplasty was performed without complications, and the patient’s vision has remained normal postoperatively.

**Fig 5:**
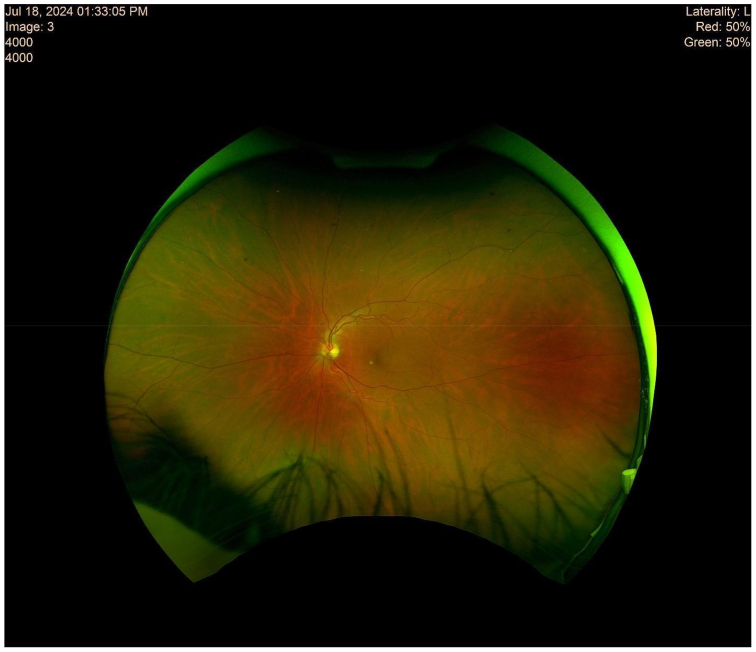
Two years after the procedure, imaging shows the normal appearance of the optic nerve and surrounding structures. The lack of edema, exudates, or significant hemorrhages reinforces the impression of a stable retinal condition postsurgery.

Follow-up imaging 2 years after the procedure revealed a normal optic nerve and stable retinal structures, with no signs of edema or hemorrhage, reinforcing the successful outcome of the surgery (Fig 5). This image shows the normal appearance of the optic nerve and surrounding structures, with the absence of edema, exudates, or significant hemorrhages, indicating a stable retinal condition postsurgery.

The patient has given their verbal consent for their images and other clinical information to be reported.

## Discussion

Atherosclerotic plaque formation in the ICA can lead to embolization, frequently affecting upstream vessels such as the ophthalmic artery, which may cause transient monocular vision loss or amaurosis fugax^3^ The feature of our clinical presentation aligns with existing body of research emphasizing how vulnerable the ophthalmic artery is to any type of stenosis in the carotid artery.^4^^,^^5^ For instance, Trago et al presented three elderly patients who experienced vision problems secondary to emboli originating from carotid artery stenosis.^6^ This further highlights the need to consider carotid artery disease in the differential diagnosis in patients with monocular vision loss.

However, it is also important to consider alternative blood circulation pathways when investigating the origin of these emboli. For example, Agarwal and his team reported a case involving a 60-year-old man who had previously experienced a complete occlusion of his right ICA and had recently undergone carotid endarterectomy for high-grade stenosis of the left ICA. This patient suddenly developed ischemia (restricted blood flow, to the retina), resulting in loss of vision shortly after. Additional examinations revealed the existence of a Hollenhorst plaque that had come from the external carotid artery, which is an important collateral pathway in the setting of ICA occlusion.^6^ This emphasizes the significance of evaluating the entire arterial pathway for individuals experiencing ocular ischemic episodes, because emboli can arise from the ICA, less often from the external carotid artery, common carotid artery, innominate artery, or even the aortic arch.

In general, our case report is unique because it presents a rare, real-time recording of an embolic event in the ophthalmic artery, documented by the patient himself, an ophthalmologist with expertise in ocular anatomy. More importantly, our case highlights the effectiveness of carotid endarterectomy in treating carotid stenosis, which is known to elevate the risk of stroke and eye-related issues such as amaurosis fugax. This finding supports existing research as internal carotid endarterectomy has been shown to both improve and prevent visual field deficits in patients with internal carotid artery stenosis.^7^ Carotid endarterectomy as the treatment of choice in the setting of symptomatic carotid stenosis for stroke prevention has been validated in multiple level 1 clinical trials.^8^^,^^9^

However, there are some limitations in our case study that need to be considered and addressed in future studies. Although the real-time recording of the embolic event is unique, this is an isolated observation and may not reflect the typical presentation or outcomes seen in broader populations. Our case emphasizes the need for thorough vascular evaluation in patients with ocular ischemic events and the critical role of timely intervention in preventing irreversible outcomes.

## Conclusions

This case sheds light on the essence of prompt diagnosis and intervention in patients with high-grade carotid stenosis presenting with transient monocular vision loss. The unique real-time recording of embolic migration in the ophthalmic artery offers a rare clinical perspective on the pathophysiology of amaurosis fugax. Although patient expertise played a pivotal role in documenting the event, the case highlights the broader need for thorough vascular evaluation and timely surgical management to prevent further cerebrovascular complications. This case adds to the growing evidence supporting carotid endarterectomy as an effective treatment in preventing stroke and preserving vision in patients with significant carotid disease.

## Funding

None.

## Disclosures

None.

## References

[1] Ismail A., Ravipati S., Gonzalez-Hernandez D. (2023). Carotid artery stenosis: a look into the diagnostic and management strategies, and related complications. Cureus.

[2] Feroze K.B., Gurnani B., O'Rourke M.C. (2024). StatPearls.

[3] Tadi P., Najem K., Margolin E. (2024). StatPearls.

[4] Bull D.A., Fante R.G., Hunter G.C. (1992). Correlation of ophthalmic findings with carotid artery stenosis. J Cardiovasc Surg (Torino).

[5] Trego M.E., Pagani J.M. (2006). Three presentations of monocular vision loss. Optometry.

[6] Agrawal A., Lazzarin S.M., Ajello D. (2023). Clinical reasoning: acute monocular vision loss in a patient with ipsilateral extracranial chronic internal carotid artery occlusion. Neurology.

[7] Konstantiniuk P., Steinbrugger I., Koter S., Muehlsteiner J., Wedrich A., Cohnert T. (2017). Impact of internal carotid endarterectomy on visual fields: a non-randomised prospective cohort study in Austria. BMJ Open.

[8] Ferguson G.G., Eliasziw M., Barr H.W. (1999). The North American symptomatic carotid endarterectomy trial. Stroke.

[9] (2004). Prevention of disabling and fatal strokes by successful carotid endarterectomy in patients without recent neurological symptoms: randomised controlled trial. Lancet.

